# Salivary biomarkers in children with juvenile idiopathic arthritis and healthy age-matched controls: a prospective observational study

**DOI:** 10.1038/s41598-022-07233-0

**Published:** 2022-02-25

**Authors:** Malin Collin, Malin Ernberg, Nikolaos Christidis, Britt Hedenberg-Magnusson

**Affiliations:** 1 Division of Oral Diagnostics and Rehabilitation, Department of Dental Medicine, Karolinska Institutet, and Scandinavian Center for Orofacial Neurosciences, 141 04 Huddinge, Sweden; 2 Department of Orofacial Pain and Jaw Function, Folktandvården Sörmland AB, Mälarsjukhuset, 611 32 Nyköping, Sweden; 3 Department of Orofacial Pain and Jaw Function, Eastman Institutet, Folktandvården Stockholms Län AB, 113 82 Stockholm, Sweden

**Keywords:** Immunology, Biomarkers, Rheumatic diseases

## Abstract

Monitoring the immune system’s regulation and signaling using saliva could be of interest for clinicians and researchers. Saliva, a biofluid with close exchange with serum, is influenced by circadian variance and oral factors such as masticatory function. This study investigated the detectability and concentration of cytokines and chemokines in saliva in children with juvenile idiopathic arthritis (JIA) as well as saliva flow and the influence of orofacial pain on saliva flow. Of the 60 participants (7–14 years old) enrolled, 30 had a diagnosis of JIA and active disease, and 30 were sex- and age-matched healthy controls. Demographic data and three validated questions regarding presence of orofacial pain and dysfunction were recorded. Stimulated whole saliva was collected and analyzed using a customized R&D bead-based immunoassay with 21 targeted biomarkers. Fourteen of these were detectable and showed similar levels in both children with JIA and controls: TNF-alpha, TNFRSF1B, MMP-2, MMP-3, IL-1alpha, IL-1beta, IL-6R alpha, IL-8, S100A8, CCL2, CCL3, IL-10, CCL11, and CXCL9. In addition, there was no difference in salivary flow rate between groups, but there was an association between orofacial pain and reduced saliva flow rate for both groups.

**Trial registration:** ClinicalTrials.gov Protocol id: 2010/2089-31/2.

## Introduction

Juvenile idiopathic arthritis (JIA), the most common rheumatic disease in children, is a heterogenous condition and an exclusion diagnosis characterized by chronic arthropathies with an onset before the age of 16^1^. Currently, the International League of Associations for Rheumatology (ILAR) divides the disease into seven subgroups based on clinical and laboratory features^1^.

Although several different pathways of activation of inflammation and tissue damage have been identified in JIA, the underlying cause of this autoimmune disease remains elusive^2^. There are apparent changes in the innate immune response causing auto-inflammation as well as a loss of immunological tolerance in the adaptive immune response, resulting in dysregulation, auto-reactive T-cells, and B-cell production of autoantibodies^2,3^. Consequently, pro-, and anti- inflammatory cytokines and chemokines play an important role in the immunopathophysiology of JIA^2,4^. Some of these cytokines and chemokines belong to the innate immune response such as TNF-alpha, interleukin-1 (IL-1), IL-6, and IL-10^5–7^, and some belong to the adaptive immune response such as the IL-2^5^. In addition, some cytokines participate in both the innate and the adaptive immune response, for example, IL-12^8^.

Changes in the expression of cytokine and chemokines can be measured in biofluids such as serum, plasma, urine, and saliva. Studies in adults and children have shown both similarities and discrepancies in cytokine expression in biofluids^9–12^. Saliva mirrors the contents of serum and with advancement made in analytical techniques it can be used for diagnostics and for disease monitoring^9–11,13^. The idea that measuring biomarkers can provide information to aid diagnosis, prognosis, and assessment of response to therapy is appealing^12^ and several studies have shown promising results^14–16^. However, there are no definitive guidelines.

Using a noninvasive sampling method such as saliva sampling to monitor disease activity in JIA could provide clinicians and researchers with valuable information^17^. However, as with blood sampling, there are diurnal variations in the saliva composition and there are functional limitations that might influence saliva flow and composition such as orofacial pain, oral hygiene, pharmacological treatment, and xerostomia^18–20^. Furthermore, previous studies have shown a high incidence of orofacial pain in children with JIA^17^ as well as divergent results regarding salivary flow rate in this group^19,21^.

Many techniques are used to collect saliva. Saliva can be collected as whole volume or as pure glandular saliva and it can be collected as stimulated or unstimulated saliva^22–24^. Different collection methods provide saliva with a noticeable difference in salivary proteome and in the relative amounts of specific proteins collected^22^. A recent study has shown that whole stimulated saliva gives a representable and acceptable picture of saliva proteins^22^. Previous studies in adults have successfully identified and measured immunological markers in saliva^11,25,26^, but few saliva studies have focused salivary biomarkers in JIA^15,19^.

In the current study three screening questions for temporomandibular disorders (3Q/TMD) were used to evaluate the impact of orofacial pain on saliva flow, and consequently, the chosen saliva collecting method. The 3Q/TMD is a validated and cost-effective screening tool for TMD that show good sensitivity and specificity to a clinical diagnosis of TMD according to the Diagnostic Criteria for TMD (DC/TMD)^27^. JIA has a high probability of temporomandibular joint (TMJ) involvement^28^, and this might influence the ability to chew, and, subsequently, saliva flow might be affected.

The present study compared saliva content regarding detectability and concentration of a panel of cytokines and chemokines in a group of children with JIA and an age-matched group of children without a JIA diagnosis from the general population. In addition, the current study investigated the salivary flow rate in children with JIA and its relation to orofacial pain. The following null hypothesis (H0) was formed: There are no differences between children with JIA and healthy controls in the expression of inflammatory mediators or in salivary flow and the salivary flow is not related to orofacial pain in children with JIA.

## Methods

### Study design and participants

This prospective case–control study was performed at the Department of Orofacial Pain and Jaw Function at the Eastman Institutet in Stockholm, Sweden between 2014 and 2017. The children with JIA were already enlisted in the Swedish national healthcare program for children with JIA at the Department of Orofacial Pain and Jaw Function at the Eastman Institutet in Stockholm, and the healthy controls were recruited from an adjoining public dental health clinic (Folktandvården Vasastan) in Stockholm. The Swedish national healthcare program (https://reuma.barnlakarforeningen.se/vardprogram/; accessed 2021-04-09) states that all children diagnosed with JIA should be referred to a dentist with special knowledge for yearly check-ups of oral and dental health and assessment of TMJ dysfunction and facial growth. In Stockholm, the Department of Orofacial Pain and Jaw Function at the Eastman Institutet in Stockholm provides this expertise. The children with JIA were also under regular supervision at the Department of Pediatric Rheumatology at Astrid Lindgren’s Children’s Hospital, Stockholm. The JIA children were diagnosed by the rheumatologist with an active disease. Some were prescribed medication, but others were not.

The following inclusion criteria were used for the JIA group: age between 7 and 14 years, a diagnosis of JIA according to ILAR^1^, and self-report of any joint symptoms such as increased pain, swelling, or functional limitations within the last 2 weeks.

The inclusion criterion for the control group was age between 7 and 14 years. Exclusion criteria for both groups were other systemic conditions or diseases that influence current or earlier inflammatory responses, diagnosis of current malignancies, intake of antibiotics the last 3 months, ongoing orthodontic treatment, or nicotine use.

Thirty children with JIA (mean (SD) age 11.1 (2.0) years) and 30 controls (11.0 (2.1)) were enrolled. The group of children with JIA consisted of 22 girls and 8 boys and the controls consisted of 19 girls and 11 boys. In the JIA group, there were four sub-diagnoses: oligoarthritis 18 (60%), polyarthritis 10 (33%), psoriatic arthritis 1 (3%), and undifferentiated JIA 1 (3%).

At time of inclusion, children in the JIA group were prescribed medications in diverse combinations: 13 (43%) were prescribed synthetic disease modifying antirheumatic drugs (sDMARDs); three (10%) were prescribed a combination of sDMARDs and biological DMARDs (bDMARDs); five (16%) were prescribed NSAIDs; and nine (30%) were not prescribed any medication. The healthy controls were not taking any medication on a regular basis at time of inclusion.

The study was performed in accordance with the Declaration of Helsinki and was approved by the Regional Ethical Review Board in Stockholm, Sweden (2010/2089-31/12) and amendment (2014/681-32). Informed permission consent was obtained from the children’s parents for participating in this study.

Since there were no previous studies on salivary biomarkers in children, the power calculation was based on a study from an adult population^29^. According to this study, inclusion of 25 participants would be sufficient to detect a statistically significant difference of 20% (SD 25%) in biomarker level between samples with a power of 80% at a significance level of 5%. To compensate for dropouts, five additional participants were included.

### Data collection

If inclusion and exclusion criteria were met and informed consent obtained, the child was enrolled in the study. The participants then had one 30 min session, at the same day as the inclusion, during which all data as well as the saliva samples were collected by two of the investigators (MC, BHM). The sessions took place at the Eastmaninstitutet for the JIA group and at Folktandvården Vasastan for the control group. Demographic data and information concerning JIA subtype and prescribed medication were obtained through the children’s medical records. To evaluate presence of orofacial pain and dysfunction the 3Q/TMD were used: Do you have pain in your temple, face, jaw, or jaw joint once a week or more? (Q1); Do you have pain once a week or more when you open your mouth or chew? (Q2); and Does your jaw lock or become stuck once a week or more? (Q3)^27,30^. Stimulated whole saliva was collected using a standardized protocol by two of the authors (MC and BHM). To avoid circadian variance, the samples were collected between 8 and 11 a.m. The participants were instructed to chew a piece of paraffin gum (Paraffin Pellets; Ivoclar Vivadent, Germany) until the gum was smooth and flexible. After this, they were instructed to swallow their saliva. Then, stimulated by chewing the same piece of paraffin gum, saliva was continuously collected for exactly 5 min^22,24^. Saliva samples collected were placed on ice and the volume was measured. The samples were then centrifuged (Centrifuge 5702; Eppendorf, Hamburg, Germany) at 1500 rpm for 15 min and the supernatant stored in 500 µl Eppendorf tubes at − 80 °C in the Biobank at Karolinska Institutet in Huddinge, Sweden pending analysis.

For the sample analyses, a customized R&D-bead based immunoassay (R&D SYSTEMS/Bio-Techne; Minneapolis, MN, US) was performed at the Plasma Profiling National Facility at the Science for Life Laboratory (SciLife) in Stockholm, Sweden using the Luminex system (#LXSAHM-21) with the following target list: Tumor necrosis factor alpha (TNF-alpha), tumor necrosis factor receptor superfamily member 1B (TNFRSF1B), Matrix metalloproteinase 1 (MMP-1), Matrix metalloproteinase 2 (MMP-2), Matrix metalloproteinase 3 (MMP-3), Matrix metalloproteinase 13 (MMP-13), Interleukin 1 alpha (IL-1 alpha), I Interleukin 1 beta (L1-beta), Interleukin 1 receptor, type II (IL-1 RII), Interleukin 2 (IL-2), Interleukin 6 (IL-6), Interleukin 6 receptor alpha (IL-6R alpha), Interleukin 8 (IL-8), Interleukin 10 (IL-10), Interleukin 12 (IL-12), C-C motif chemokine ligand 2 (CCL2), C-C motif chemokine ligand 3 (CCL3), C-C motif chemokine ligand 11 (CCL11), C-C motif chemokine ligand 22 (CCL22), C-X-C motif chemokine ligand 9 (CXCL9), and S100 calcium-binding protein A8 (S100A8) (Suppl. Table 1). These markers were chosen by the authors based on a literature search of cytokines and chemokines that have been implicated in the pathophysiology of inflammatory joint diseases^2,3,9,12,16,31,32^. A pilot test showed that 14 of these 21 targeted proteins had dilution-depending curves at a dilution of 1:2 and that seven were compromised for matrix effects: MMP-1, MMP-13, IL-1 RII, IL-2, IL-6, IL-12, and CCL22. These markers were therefore excluded from further analyses, i.e. the main analysis was performed based on the remaining 14 proteins.

### Statistics

For the statistical analyses, the data were analyzed in R Core Team (R Core Team, version 3.2.6; R Foundation for Statistical Computing; Austria) and IBM SPSS Statistics 25 (IBM SPSS Statistics for Windows, Version 23.0; IBM, NY, USA) For all tests, the level of significance was set to p < 0.050. Mean, standard deviation (SD), and percent (%) were used for descriptive statistics regarding age, sex, medication, and type of diagnosis, and median and interquartile range were used for descriptive statistics regarding protein levels. The Shapiro–Wilk test was used to test for normality, and the skewedness of the variables were assessed.

Saliva flow rate in association with group, type of diagnosis, age, gender, medication, and 3Q/TMD were tested using t-test (Welch adjustment for two groups) and ANOVA (for > 2 groups). For the 3Q/TMD, associations were tested using Fisher’s exact test. Linear regression model was applied only for age association. All inflammatory markers showed a skewed distribution and data were log-transformed for the analysis of distribution of protein values (Table 2; Fig. 2). T-test without any adjustments was applied in the analysis of association with group, and linear regression model with adjustment was applied for association with age. For all other variables associated with levels of biomarkers, ANOVA and Benjamini–Hochberg procedure was used^33^.

## Results

### Orofacial pain and dysfunction

**Figure 1:**
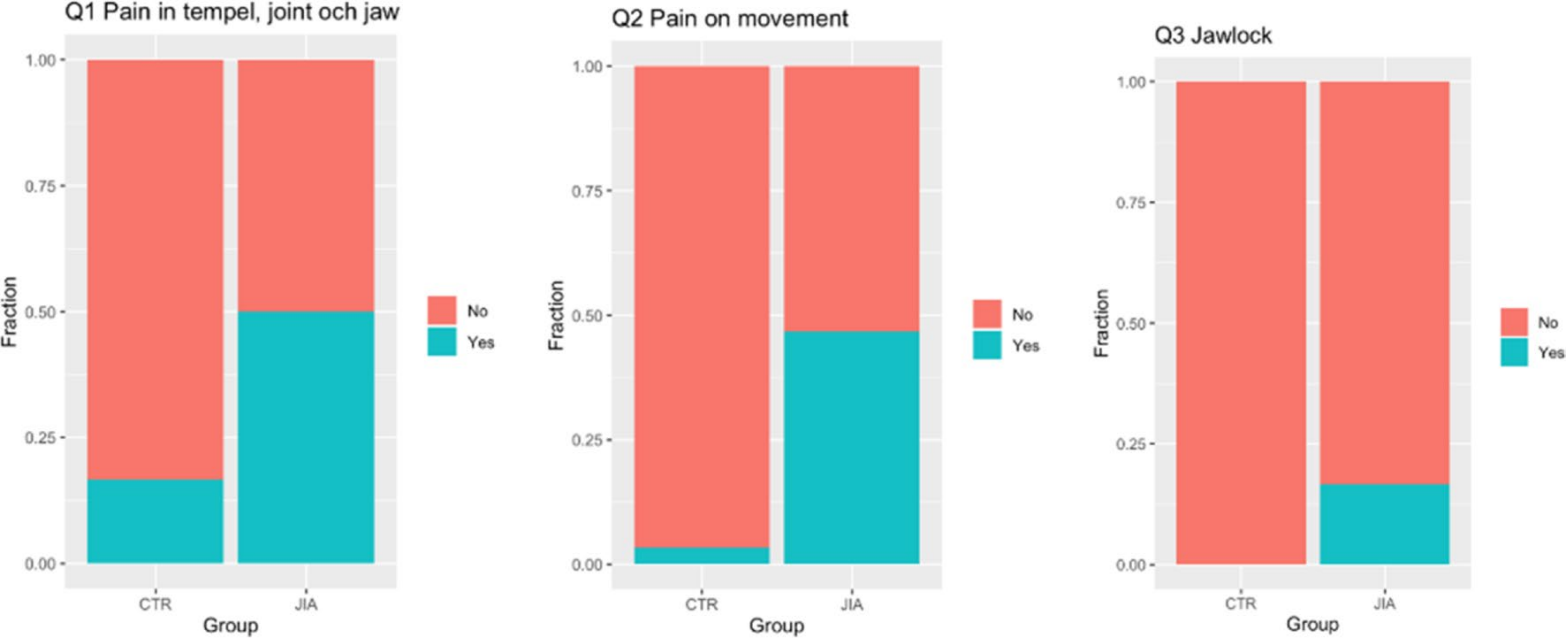
This figure shows the distribution of temporomandibular symptoms according to the 3Q/TMD questions in the 30 children with a diagnosis of JIA and in the 30 healthy controls. The JIA group had significantly more orofacial and functional pain (Q1 and Q2).

The 3Q/TMD showed that the children with JIA had more pain (Q1, Q2) and functional disturbances (Q3) than the healthy controls. The presence of positive answers to Q1 and Q2 was significantly higher in the children with JIA than in the controls (p = 0.010 and p < 0.001, respectively), and there was a trend to a significant difference for Q3 (p = 0.052). These results indicate that the children with JIA to larger extent suffer from orofacial pain and dysfunction (Fig. 1).

### Saliva flow rate

There was no difference in mean (SD) saliva flow rate between groups. In the children with JIA, the rate was 0.99 (0.41) ml/min; in the controls, the rate was 1.05 (0.46) ml/min. Neither age, gender, nor type of medication influenced saliva flow rate. However, participants with a positive answer to Q1 of 3Q/TMD, irrespective of group, had a significantly lower saliva flow rate (p = 0.026).

### Protein analyses

**Figure 2:**
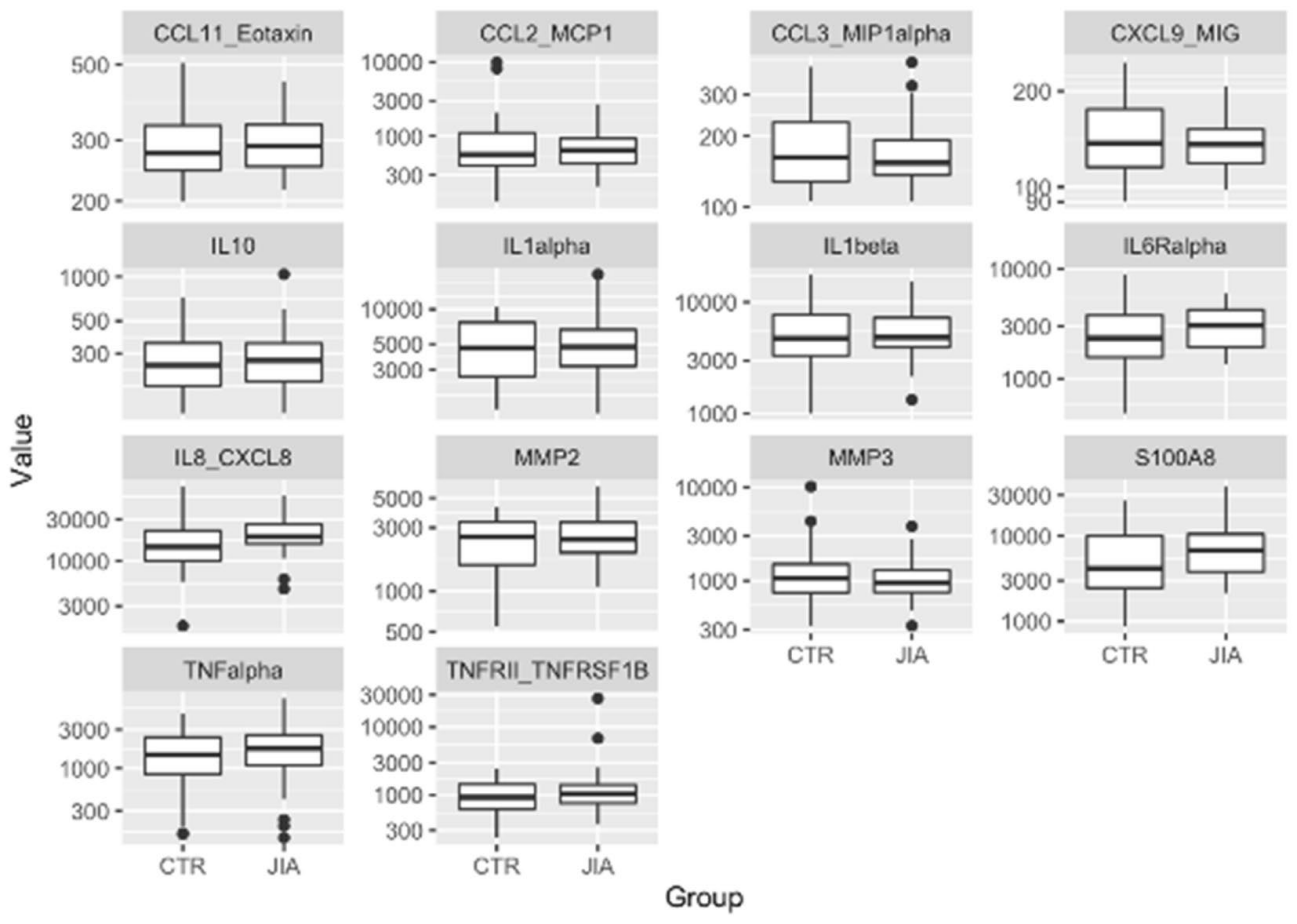
The figure shows the distribution of protein values in saliva in 30 children with a diagnosis of JIA and in 30 healthy controls (CTR). There was no significant difference in levels between the two groups in any of the measured proteins.

The customized main immunoassay analysis measured the following: TNF-alpha, TNFRSF1B, MMP-2, MMP-3, IL-1alpha, IL-1beta, IL-6R alpha, IL-8, S100A8, CCL2, CCL3, IL-10, CCL11, and CXCL9 (Suppl. Table 1). In this material, there was no significant difference in biomarker levels between the two groups (p > 0.050), but IL-8 showed a tendency (p = 0.063) to higher levels in the participants with JIA (Table 1; Fig. 2).

**Table 1:** Mean (SD) of protein concentration (pg/mL) in saliva of children with juvenile idiopathic arthritis (JIA) and healthy controls (CTR).

pg/mL	JIA		CTR		P-value
	Mean (SD)	Median [min, max]	Mean (SD)	Median [min, max]	
TNF-alpha	7.31 (1.01)	7.47 [4.93, 8.89]	7.07 (1.06)	7.29 [5.04, 8.47]	0.371
MMP-3	6.93 (0.53)	6.85 [5.79, 8.25]	7.11 (0.74)	6.97 [5.80, 9.23]	0.283
IL-8/CXCL8	9.85 (0.51)	9.86 [8.46, 10.90]	9.55 (0.69)	9.57 [7.48, 11.20]	0.063
MMP-2	7.83 (0.41)	7.80 [6.99, 8.71]	7.68 (0.60)	7.84 [6.31, 8.36]	0.235
S100A8	8.81 (0.67)	8.82 [7.67, 10.50]	8.47 (0.91)	8.34 [6.79, 10.20]	0.104
IL-10	5.61 (0.46)	5.59 [4.76, 6.95]	5.53 (0.45)	5.52 [4.76, 6.59]	0.478
CCL2/MCP-1	6.44 (0.64)	6.47 [5.34, 7.90]	6.47 (0.99)	6.33 [4.87, 9.20]	0.890
IL-6R alpha	7.97 (0.46)	8.02 [7.21, 8.70]	7.74 (0.62)	7.76 [6.17, 9.10]	0.117
IL-1 beta	8.54 (0.54)	8.48 [7.20, 9.64]	8.54 (0.68)	8.47 [6.91, 9.78]	0.982
CCL3/MIP-1 alpha	5.13 (0.35)	5.03 [4.65, 6.02]	5.18 (0.40)	5.09 [4.66, 5.98]	0.586
IL-1 alpha	8.49 (0.69)	8.46 [7.13, 9.91]	8.34 (0.62)	8.44 [7.22, 9.26]	0.390
CXCL9/MIG	4.93 (0.21)	4.91 [4.58, 5.33]	4.96 (0.31)	4.92 [4.50, 5.51]	0.669
TNFRSF1B RII/TNFRSF1B	7.07 (0.82)	6.95 [5.90, 10.2]	6.78 (0.65)	6.83 [5.45, 7.81]	0.142
CCL11/eotaxin	5.67 (0.18)	5.67 [5.37, 6.10]	5.69 (0.25)	5.62 [5.29, 6.23]	0.742

No association was observed in the linear regression model with adjustment for age and gender (unadjusted P < 0.05).

**Figure 3:**
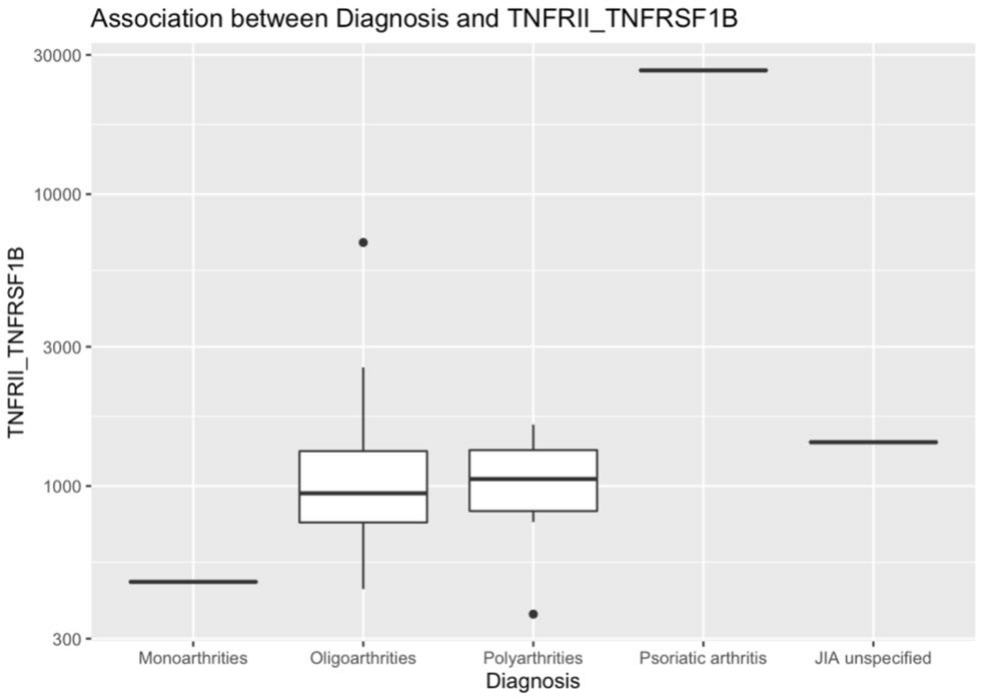
This figure illustrates the association between protein levels of TNFRSF1B (tumor necrosis factor receptor superfamily member 1B) and the different sub-diagnoses of JIA in 30 children with JIA: Only psoriatic arthritis could reveal a significant association.

In the JIA group, TNFRSF1B (TNFRII-TNFRSF1B) was significantly associated with a diagnosis of psoriatic arthritis (p < 0.050) according to the Benjamini–Hochberg procedure (Table 2). This association was driven by one outlier (Fig. 3). Neither age, sex, nor medication influenced the levels of biomarkers in any group (linear regression adjusted for age and gender).

**Table 2:** Associations between protein levels in saliva and age, gender, sub-diagnosis, as well as medication in children with juvenile idiopathic arthritis (JIA).

Protein	Age	Gender	Diagnosis	Medication
TNFalpha	0.831	0.502	0.998	0.640
MMP3	0.587	0.673	0.998	0.640
IL8_CXCL8	0.878	0.867	0.998	0.103
MMP2	0.588	0.502	0.998	0.640
S100A8	0.579	0.673	0.998	0.713
IL10	0.579	0.502	0.998	0.615
CCL2_MCP1	0.579	0.939	0.998	0.139
IL6Ralpha	0.831	0.528	0.998	0.274
IL1beta	0.587	0.867	0.998	0.615
CCL3_MIP1alpha	0.576	0.528	0.998	0.425
IL1alpha	0.588	0.867	0.998	0.713
CXCL9_MIG	0.576	0.528	0.998	0.343
TNFRSF1B	0.831	0.867	**0.004**	0.274
CCL11_Eotaxin	0.579	0.502	0.998	0.574

FDRs are presented for the statistically significant association (FDR < 0.05 by Benjamini–Hochberg procedure). TNFRSF1B was significantly associated with diagnosis in the JIA group.

Significant values are in bold.

**Figure 4:**
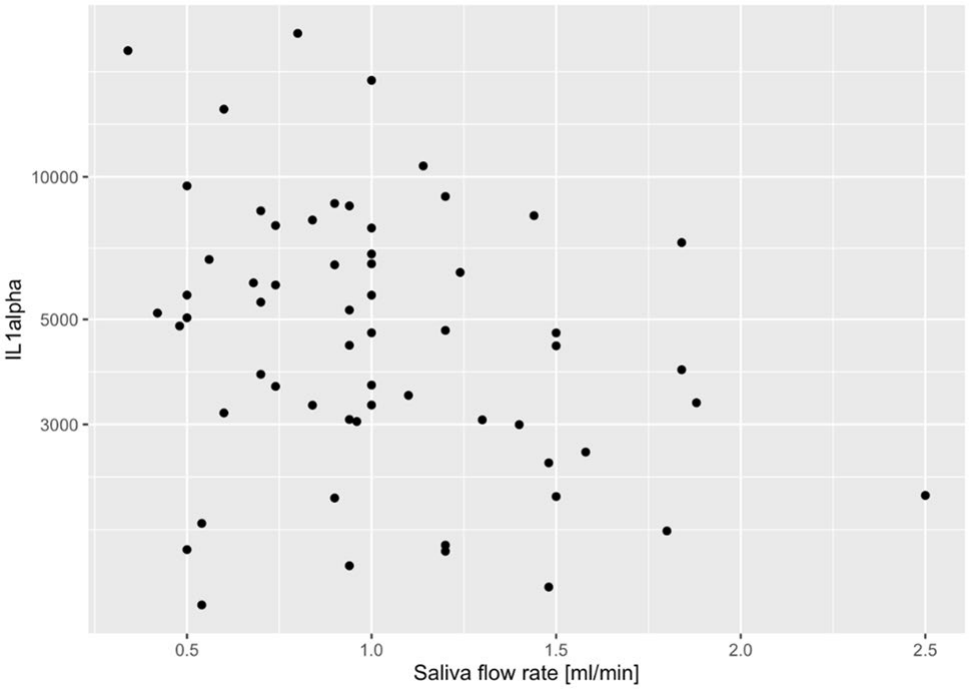
The figure shows the distribution of IL1-alfa (Interleukin-1alpha) in the saliva of 30 children with a diagnosis of JIA and in the 30 healthy controls (CTR) together (n = 60). IL1alpha showed indication of the association with saliva flow (unadjusted p < 0.050).

When the two sample groups were combined, IL-1 alpha showed a negative association with saliva flow rate (p = 0.025) independent of diagnosis (JIA or CTR) and JIA sub-diagnosis (Fig. 4).

## Discussion

The main findings from the current study were that there were no differences in expressed levels of pro- and anti-inflammatory salivary markers or in salivary flow between children with a diagnosis of JIA and healthy controls, and no association between orofacial pain and reduced saliva flow rate in JIA. Hence, the null-hypotheses were accepted.

Our pilot test, which was used to validate the immunoassay (R&D systems), showed matrix effects for seven of 21 targeted biomarkers, i.e., they could not be detected in saliva with the method used. The main analyses were adjusted accordingly. This approach is in line with previous studies in adults and children that show deviating findings regarding coherence and differences in the detectability and levels of inflammatory markers in body fluids in inflammatory diseases^9–12^. Some of the proteins detected have been found in saliva in adults with rheumatoid disease such as IL-10, and several have been detected in saliva in other diseases^9^. Although this study did not detect IL-2 and IL-6, other studies have detected IL-2 and IL-6 using ELISA and multiplex (bead) analyses of saliva^9,11^. The cause of these discrepancies could be differences in sampling technique, or our choice of immunoassay and combination of biomarkers used for the analyses. Nevertheless, we conclude that in this setting most of the investigated biomarkers were detectable by the applied method.

The main analysis of the remaining 14 proteins showed no significant differences in saliva concentration between children with JIA and the controls. IL-8 had the closest association with a diagnosis of JIA (p = 0.06). Our data also show that a high concentration of TNFRSF1B in saliva was associated with a diagnosis of juvenile psoriatic arthritis (p = 0.004), but the association was based on a single individual, so no definitive conclusion can be drawn (Fig. 3). Taken together, these findings indicate a need for studies with a larger number of patients in all subgroups of JIA as there might be differences in cytokine profiles between them^12,34^.

We used paraffin to stimulate whole saliva as this method gives an adequate picture of saliva content^22^. The collecting technique used for saliva sampling worked well in the age group 7–14 years. Using stimulated saliva increases the risk of contamination from gingival fluid and dental plaque, and this risk is higher in children with JIA as they suffer from gingival inflammation and poor dental health to a larger extent than healthy children^18,35^. Nevertheless, in the current study, there were no differences in the expression of biomarkers in saliva between the two groups. If the saliva was contaminated, this contamination would have been the same in both groups and therefore would not have affected the outcome of the study.

There was no difference in stimulated whole saliva flow rate observed between the two groups. Furthermore, there was no association between saliva flow rate and age, sex, or medication. This finding is in accordance with a previous study, also performed in Sweden^18^, that indicated there are no variances in either stimulated or unstimulated salivary flow rate between children with JIA and controls. In contrast, a few studies show a reduction in unstimulated and stimulated saliva flow rate in children with JIA^19,21^. Altogether, these findings suggest this discrepancy could be due to disease activity, methodological differences, time of salivary collections, or the small size of the study populations.

The influence of orofacial pain on saliva flow rate assessed using the 3Q/TMD showed an association between presence of orofacial pain (Q1) and a lower saliva flow rate, whereas the other two questions had no impact. Even if there was no association between presence of orofacial pain and saliva flow rate in JIA, saliva flow rate was lower in children who had answered yes to Q1, independent of diagnosis. This finding indicates that the decrease in salivary flow is due to pain in the orofacial region rather than due to JIA disease, a finding that agrees with previous studies that demonstrate a reduction in salivary flow in an adult population suffering from painful TMD^20,36^. These are findings from just a few studies; however, one study indicates that there are no differences^37^. To our knowledge, our study is the first study to show that orofacial pain influences saliva flow rate in children in general.

There was a significant difference in self-reported orofacial pain according to the 3Q/TMD between the controls and children with JIA. In children with JIA, 50% reported resting pain or pain on movement in the temporomandibular area. Similarly, earlier studies have reported a high degree of orofacial pain in JIA^17^. It is well established that the TMJ might be involved in JIA and be the source of pain and functional limitations in the orofacial area^38^. Some other factors that predict onset of orofacial pain in children are the number of preexisting pain conditions, female gender, and psychosocial load^39^. All these factors are more common in children with JIA than in healthy children, and this difference could explain the differences between the groups^1,40^.

The current study has several strengths, including its use of a representative group of children with JIA regarding distributions of sex, JIA diagnosis, and medication, a matched control group, a standardized saliva collecting technique, and a validation of the immunoassay^1^. A limitation is the small sample size, which might lead to a type II error especially when looking at the JIA subtypes. As this study is an exploratory study, future studies should consider sub-diagnoses of JIA. The small sample size as well as the degree of medication in the children with JIA could explain why there were no differences in inflammatory markers between the groups. This lack of difference was evident even though active disease was an inclusion criterion for the children with JIA and the initial power calculation indicated that the sample size was sufficient. Another limitation could be that processing of the saliva samples might have removed or damaged some proteins. However, if this were the case, both groups would have been equally affected.

In conclusion, our exploratory study showed no differences between children with JIA and healthy children in the expression of certain salivary cytokines and chemokines or salivary flow rate. However, orofacial pain seemed to affect salivary flow in children and adolescents independent of the presence or absence of JIA. As the finding indicates differences in biomarker profile in saliva between the JIA subgroups, future studies should consider including a sufficient number of participants in each JIA subgroup.

## Supplementary Information

Supplementary Table 1.

## Data Availability

The data that support the findings of the study are available from the corresponding author upon reasonable request.

## References

[1] Petty RE (2004). International league of associations for rheumatology classification of juvenile idiopathic arthritis: Second revision, Edmonton, 2001. J. Rheumatol..

[2] Zaripova LN (2021). Juvenile idiopathic arthritis: From aetiopathogenesis to therapeutic approaches. Pediatr. Rheumatol. Online J..

[3] Spierings J, van Eden W (2017). Heat shock proteins and their immunomodulatory role in inflammatory arthritis. Rheumatology.

[4] Cohen S (1976). Cell mediated immunity and the inflammatory system. Hum. Pathol..

[5] Szondy Z, Pallai A (2016). Transmembrane TNF-alpha reverse signaling leading to TGF-beta production is selectively activated by TNF targeting molecules: Therapeutic implications. Pharmacol. Res..

[6] Dinarello CA, Savage N (1989). Interleukin-1 and its receptor. Crit. Rev. Immunol..

[7] Caiello I (2014). IL-6 amplifies TLR mediated cytokine and chemokine production: Implications for the pathogenesis of rheumatic inflammatory diseases. PLoS ONE.

[8] Wang KS, Frank DA, Ritz J (2000). Interleukin-2 enhances the response of natural killer cells to interleukin-12 through up-regulation of the interleukin-12 receptor and STAT4. Blood.

[9] Khan A (2012). Detection and quantitation of forty eight cytokines, chemokines, growth factors and nine acute phase proteins in healthy human plasma, saliva and urine. J. Proteomics..

[10] Williamson S, Munro C, Pickler R, Grap MJ, Elswick RK (2012). Comparison of biomarkers in blood and saliva in healthy adults. Nurs. Res. Pract..

[11] Sikorska D (2018). Diagnostic value of salivary CRP and IL-6 in patients undergoing anti-TNF-alpha therapy for rheumatic disease. Inflammopharmacology.

[12] de Jager W (2007). Blood and synovial fluid cytokine signatures in patients with juvenile idiopathic arthritis: A cross-sectional study. Ann. Rheum. Dis..

[13] Dawes C, Wong DTW (2019). Role of saliva and salivary diagnostics in the advancement of oral health. J. Dent. Res..

[14] Patwardhan, A. The utility and experience with disease biomarkers in juvenile onset arthritis vs. adult onset arthritis. *Cureus.* **11**, e5131, 5131–5142, 10.7759/cureus.5131 (2019). 10.7759/cureus.5131 PMC6649876 31341750

[15] Brik R (2010). Salivary antioxidants and metalloproteinases in juvenile idiopathic arthritis. Mol. Med..

[16] Kaminiarczyk-Pyzalka D (2014). Proinflammatory cytokines in monitoring the course of disease and effectiveness of treatment with etanercept (ETN) of children with oligo- and polyarticular juvenile idiopathic arthritis (JIA). Clin. Lab..

[17] Leksell E, Ernberg M, Magnusson B, Hedenberg-Magnusson B (2012). Orofacial pain and dysfunction in children with juvenile idiopathic arthritis: A case-control study. Scand. J. Rheumatol..

[18] Leksell E, Ernberg M, Magnusson B, Hedenberg-Magnusson B (2008). Intraoral condition in children with juvenile idiopathic arthritis compared to controls. Int. J. Paediatr. Dent..

[19] Kobus A (2017). Unstimulated salivary flow, pH, proteins and oral health in patients with Juvenile Idiopathic Arthritis. BMC Oral Health.

[20] da Silva LA, Teixeira MJ, de Siqueira JT, de Siqueira SR (2011). Xerostomia and salivary flow in patients with orofacial pain compared with controls. Arch. Oral Biol..

[21] Walton AG, Welbury RR, Foster HE, Wright WG, Thomason JM (2002). Sialochemistry in juvenile idiopathic arthritis. Oral Dis..

[22] Jasim H, Olausson P, Hedenberg-Magnusson B, Ernberg M, Ghafouri B (2016). The proteomic profile of whole and glandular saliva in healthy pain-free subjects. Sci. Rep..

[23] Stephen KW, Speirs CF (1976). Methods for collecting individual components of mixed saliva: The relevance to clinical pharmacology. Br. J. Clin. Pharmacol..

[24] Feres de Melo AR, Ferreira de Souza A, de Oliveira Perestrelo B, Leite MF (2014). Clinical oral and salivary parameters of children with juvenile idiopathic arthritis. Oral Surg. Oral Med. Oral Pathol. Oral Radiol..

[25] Rhodus NL (2005). The feasibility of monitoring NF-kappaB associated cytokines: TNF-alpha, IL-1alpha, IL-6, and IL-8 in whole saliva for the malignant transformation of oral lichen planus. Mol. Carcinogen..

[26] Svard A, Kastbom A, Sommarin Y, Skogh T (2013). Salivary IgA antibodies to cyclic citrullinated peptides (CCP) in rheumatoid arthritis. Immunobiology.

[27] Lövgren A (2016). Validity of three screening questions (3Q/TMD) in relation to the DC/TMD. J. Oral Rehabil..

[28] Collin M, Hagelberg S, Ernberg M, Hedenberg-Magnusson B, Christidis N (2022). Temporomandibular joint involvement in children with juvenile idiopathic arthritis-symptoms, clinical signs and radiographic findings. J. Oral Rehabil..

[29] Jasim H, Carlsson A, Hedenberg-Magnusson B, Ghafouri B, Ernberg M (2018). Saliva as a medium to detect and measure biomarkers related to pain. Sci. Rep..

[30] Nilsson IM (2007). Reliability, validity, incidence and impact of temporormandibular pain disorders in adolescents. Swed. Dent. J. Suppl..

[31] Idriss HT, Naismith JH (2000). TNF alpha and the TNF receptor superfamily: Structure–function relationship(s). Microsc. Res. Tech..

[32] Palomo J, Dietrich D, Martin P, Palmer G, Gabay C (2015). The interleukin (IL)-1 cytokine family–balance between agonists and antagonists in inflammatory diseases. Cytokine.

[33] Hochberg Y, Benjamini Y (1990). More powerful procedures for multiple significance testing. Stat. Med..

[34] De Benedetti F, Ravelli A, Martini A (1997). Cytokines in juvenile rheumatoid arthritis. Curr. Opin. Rheumatol..

[35] Kobus A, Baginska J, Lapinska-Antonczuk J, Lawicki S, Kierklo A (2019). Levels of selected matrix metalloproteinases, their inhibitors in saliva, and oral status in juvenile idiopathic arthritis patients vs. healthy controls. Biomed. Res. Int..

[36] de Andrade CM (2019). Salivary biomarkers for caries susceptibility and mental stress in individuals with facial pain. Cranio.

[37] Mladenovic I, Krunic J, Stojanovic N, Markovic D, de Siqueira SR (2018). Limited jaw movements and somatization (but not pain) may play a role in salivary flow in female patients with temporomandibular disorders. J. Oral Facial Pain Headache..

[38] Stoustrup P (2017). Clinical orofacial examination in juvenile idiopathic arthritis: International consensus-based recommendations for monitoring patients in clinical practice and research studies. J. Rheumatol..

[39] LeResche L, Mancl LA, Drangsholt MT, Huang G, Von Korff M (2007). Predictors of onset of facial pain and temporomandibular disorders in early adolescence. Pain.

[40] Leksell E, Hallberg U, Magnusson B, Ernberg M, Hedenberg-Magnusson B (2015). Perceived oral health and care of children with juvenile idiopathic arthritis: A qualitative study. J. Oral Facial Pain Headache..

