# Supplementary material for: Salivary biomarkers in children with juvenile idiopathic arthritis and healthy age-matched controls: a prospective observational study
Source: Sci Rep. 2022 Feb 25;12:3240. doi: 10.1038/s41598-022-07233-0 (PMC8881454; doi:10.1038/s41598-022-07233-0)
Supplement: Supplementary file 1 — Supplementary Table 1. [file 41598_2022_7233_MOESM1_ESM.docx]

**Suppl. Table 1.** Abbreviations and names of proteins in text, tables, and figures

| TNF-alpha | Tumor necrosis factor alpha |
| --- | --- |
| TNFRSF1B | Tumor necrosis factor receptor superfamily member 1B |
| MMP-1 | Matrix metalloproteinase 1 |
| MMP-2 | Matrix metalloproteinase 2 |
| MMP-3 | Matrix metalloproteinase 3 |
| MMP-13 | Matrix metalloproteinase 13 |
| IL-1alpha | Interleukin 1 alpha |
| IL-1beta | Interleukin 1 beta |
| IL-1 RII | Interleukin 1 receptor, type II |
| IL-2 | Interleukin 2 |
| IL-6 | Interleukin 6 |
| IL-6R alpha | Interleukin 6 receptor alpha |
| IL-8 | Interleukin 8 |
| IL-10 | Interleukin 10 |
| IL-12 | Interleukin 12 |
| CCL2 | C-C motif chemokine ligand 2 |
| CCL3 | C-C motif chemokine ligand 3 |
| CCL22 | C-C motif chemokine ligand 22 |
| CCL11 | C-C motif chemokine Ligand 11 |
| CXCL9 | C-X-C motif chemokine ligand 9 |
| S100A8 | S100 calcium-binding protein A8 |
